# Isolation, Characterization, and Genomic Analysis of Three Novel *E. coli* Bacteriophages That Effectively Infect *E. coli* O18

**DOI:** 10.3390/microorganisms10030589

**Published:** 2022-03-09

**Authors:** Fatma Abdelrahman, Nouran Rezk, Mohamed S. Fayez, Mohamed Abdelmoteleb, Reham Atteya, Mohamed Elhadidy, Ayman El-Shibiny

**Affiliations:** 1 Center for Microbiology and Phage Therapy, Zewail City of Science and Technology, Giza 12578, Egypt; fabdelrahman@zewailcity.edu.eg (F.A.); p-nrezk@zewailcity.edu.eg (N.R.); p-mfayez@zewailcity.edu.eg (M.S.F.); 2 Department of Botany, Faculty of Science, Mansoura University, Mansoura 35516, Egypt; dmotelb87@gmail.com; 3 Center for Genomics, Helmy Institute for Medical Science, Zewail City of Science and Technology, Giza 12578, Egypt; p-rattaeya@zewailcity.edu.eg; 4 Biomedical Sciences Program, University of Science and Technology, Zewail City of Science and Technology, October Gardens, 6th of October City, Giza 12578, Egypt; melhadidy@zewailcity.edu.eg; 5 Department of Bacteriology, Mycology and Immunology, Faculty of Veterinary Medicine, Mansoura University, Mansoura 35516, Egypt; 6 Faculty of Environmental Agricultural Sciences, Arish University, Arish 45511, Egypt

**Keywords:** *Escherichia coli*, avian pathogenic *E. coli* (APEC), bacteriophages, *Siphoviridae*

## Abstract

*Escherichia coli* (*E. coli*) is one of the most common pathogenic bacteria worldwide. Avian pathogenic *E. coli* (APEC) causes severe systemic disease in poultry (Colibacillosis), and accordingly, has an extreme risk to the poultry industry and public health worldwide. Due to the increased rate of multi-drug resistance among these bacteria, it is necessary to find an alternative therapy to antibiotics to treat such infections. Bacteriophages are considered one of the best solutions. This study aimed to isolate, characterize, and evaluate the potential use of isolated bacteriophages to control *E. coli* infections in poultry. Three novel phages against *E. coli* O18 were isolated from sewage water and characterized in vitro. The genome size of the three phages was estimated to be 44,776 bp, and the electron microscopic analysis showed that they belonged to the *Siphoviridae* family, in the order Caudovirales. Phages showed good tolerance to a broad range of pH and temperature. The complete genomes of three phages were sequenced and deposited into the GenBank database. The closely related published genomes of *Escherichia* phages were identified using BLASTn alignment and phylogenetic trees. The prediction of the open reading frames (ORFs) identified protein-coding genes that are responsible for functions that have been assigned such as cell lysis proteins, DNA packaging proteins, structural proteins, and DNA replication/transcription/repair proteins.

## 1. Introduction

*E. coli* is the most thoroughly studied bacterial species because it can be easily propagated and genetically manipulated. It has highly pathogenic strains and causes many types of infections. In poultry as well as in humans, *E. coli* lives in the lower digestive tract and colonizes in the first 24 h after hatching [1]. Even though many strains are harmless, some have developed the ability to cause severe intestinal and extraintestinal diseases. Extraintestinal pathogenic *E. coli* (ExPEC) strains are subclassified based on the host and the site of infection into some variants; neonatal meningitis *E. coli* (NMEC), sepsis-associated *E. coli* (SEPEC), and uropathogenic *E. coli* (UPEC) that cause urinary tract infections (UTI), and avian pathogenic *E. coli* strains (APEC) that infect birds of all ages, thus affecting all types of poultry production, causing septicemia, polyserositis, respiratory tract infections (RTI), cellulitis, salpingitis, and others, commonly called colibacillosis [2,3].

Avian colibacillosis harms the poultry industry, causing severe economic losses reaching hundreds of millions of dollars worldwide, with high rates of morbidity and mortality (up to 20%) in poultry [4] and up to 53.5% in young chickens [5].

The relationship between the drug-resistant human *E. coli* strains and those found in poultry has been reported by James R. Johnson et al. (2007), with drug-susceptible human and poultry isolates included in the study. They revealed that many drug-resistant human *E. coli* isolates might have originated from poultry. In contrast, drug-resistant poultry *E. coli* isolates have likely been derived from susceptible poultry *E. coli* strains by conversion to resistance triggered under the selection pressure from overuse/misuse of antimicrobial drugs by poultry farmers [6]. Another study by Jakobsen et al. showed that the ExPEC related virulence gene content of *E. coli* from UTI patients and meat products matched their virulence and antibiotic resistance [7].

The great diversity among APEC strains limits vaccination possibilities [4], and antibiotics were an option until the emergence of antibiotic-resistant strains. As reported in a study by Halfaoui et al. (2017), fifty *E. coli* strains isolated from poultry revealed 94.12% phenotypic resistance to tetracyclines, 91.5% to flumequine, 88.89% to sulfamethoxazole-trimethoprim, 86.27% to enrofloxacin, 85.62% to nalidixic acid, 83.01% to ampicillin, and 75.81% to doxycycline [8]. Furthermore, the World Health Organization (WHO) has predicted, bearing in mind the rise in antibiotic resistance and scarcity of antibiotic production, that by 2050, we will face the same scenario as in the pre-penicillin era [9].

Therefore, alternatives to antibiotics and vaccines are necessary, and bacteriophages (phages) are among such options.

Before discovering antibiotics, many studies were conducted to test the efficacy of lytic phages in treating bacterial infections. Phages have many advantages over antibiotics. Phages are natural bacterial killers and are considered the most abundant microbial entities; about 10^31^ phage virions are present on Earth [10]. In addition, there are some extra advantages of phage therapy compared to antibiotics as phages are naturally available in the environment, and are easy and cheap to produce [11]. Moreover, they are particular to bacterial targets without adverse impacts on normal microbiota as antibiotics do [12,13]. The studies on bacteriophages stopped, though, except for the former Soviet Union and Eastern Europe, due to the widespread availability of antibiotics at that time [14].

Many previous studies on the application of bacteriophages in veterinary medicine reported the successful use of bacteriophages in treating farm animal pathogens such as *E. coli* [15,16,17], *Salmonella* spp. [18,19,20], and *Campylobacter* spp. [21,22]. Therefore, isolation and characterization of new phages is potentially useful for phage therapy applications in both human and veterinary pathogens.

In this study, we characterized and evaluated the potential use of the newly isolated bacteriophages (ZCEC10, ZCEC11, and ZCEC12) to control *E. coli* O18 infections in poultry as a keystone for finding a possible alternative treatment of these pathogens.

## 2. Materials and Methods

### 2.1. Bacterial Strains and Growth Conditions

The bacterial host *E. coil* O18 and other *E. coli* serotypes used in this study were provided by MEVAC Company, Egypt to the Zewail City of Science, Technology, and Innovation’s Center for Microbiology and Phage therapy (CMP). Isolates were preserved in tryptone soy broth (TSB; Oxoid, UK) containing 20% (*w*/*v*) glycerol at −80 °C for further use.

### 2.2. Antimicrobial Susceptibility Testing

The antibiotic susceptibility of *E. coli* O18 strain was determined through the disc diffusion method [23] using ten commercially available antibiotics (Oxoid, UK) to gentamicin (10 µg), aztreonam (30 µg), amoxicillin/clavulanic acid (30 µg), ampicillin (10 µg), amikacin (30 µg), cefoxitin (30 µg), ampicillin/sulbactam (20 µg), cefotaxime (30 µg), tetracycline (30 µg), and ceftriaxone (30 µg) as indicated in Table 1. In brief, 100 µL of the bacterial culture was spread over the tryptone soy agar (TSA) plate, followed by the impregnation of the antibiotic discs on it, and incubated at 37 °C for 24 h. The diameter of the inhibition zones was measured, and the results were interpreted based on the guidelines of the Clinical and Laboratory Standards Institute [24].

### 2.3. 16S Ribotyping

16S rRNA gene amplification and sequencing using 16S rRNA universal primers 27F (5′-AGAGTTTGATCCTGGCTCAG-3′) and 1492R (5′-GGTTACCTTGTTACGACTT-3′) were used to identify *E. coli* O18. An automated fluorescent-DNA sequencer (Applied Biosystem, model 373A/USA) was used to identify the 16S rRNA sequence. Finch TV software (https://digitalworldbiology.com/FinchTV/; Access date: 3 October 2021) was used to process the sequences acquired. To detect homology, researchers used BLASTN: Basic Local Alignment Search Tool (http://www.ncbi.nlm.nih.gov/BLAST/Blast.cgi/; Access date: 3 October 2021) against the 16S ribosomal RNA database. Under the entry number OK355402, the sequence was deposited in the NCBI GenBank database.

### 2.4. Phage Selection, Isolation, Purification, and Amplification

Bacteriophages were isolated from sewage at a hospital. For phage isolation, *E. coli* O18 was employed as a bacterial host. As previously documented, phages were isolated from single plaque isolates [25]. To isolate pure phage stocks, phage plaques were purified by picking up a single phage plaque with sterile micropipette tips and repeating the process at least five times. To achieve higher phage stocks, the isolated phages were enriched and propagated as follows: in TSB, an indicator host (100 mL, 107 CFU/mL) was infected separately with each phage and incubated at 37 °C with 120 rpm shaking. Next, the lysates were centrifuged at 6000× *g* for 15 min at 4 °C to eliminate any leftover bacterial cells and debris. The phage-containing supernatant was then centrifuged for one hour at 4 °C at 15,000× *g*. Finally, the phage pellets were resuspended in SM buffer (100 mM MgSO4/7H2O; 10 mM NaCl; 50 mM Tris-HCl; pH 7.5) and filtered using 0.22 m syringe filters. A double-layer agar method was used to determine the phage titers and confirm the presence of lytic phages in the filtrate [26,27].

### 2.5. Host Range Determination

The host range of the various phages was examined using the spot assay method against a variety of *E. coli* and *Salmonella* spp. strains, as previously described [28]. Clear plaques revealed high host specificity after incubation; turbid or no plaques revealed non-infectivity. The bacterial strains used for the host range determination are listed in Table 2.

### 2.6. The Influence of Temperature and pH on Bacteriophages Stability

The temperature stability of phages ZCEC10, ZCEC11, and ZCEC12 (10^9^ PFU/mL) was assessed in a water bath at −20, 4, 37, 50, 60, 70, 75, and 80 °C after one-hour incubation [29]. To assess phage titer, successive dilutions of the phage were spotted three times on a lawn of host strain *E. coli* O18 using a double-layer agar overlay method immediately after incubation [30]. The phage titers of the previously stated phages were determined by incubating them for one hour at varied pH values of 2, 3, 4, 5, 7, 9, 10, 11, and 12 [31]. The pH values were adjusted in the SM phage buffer [32].

### 2.7. Time Killing Curve of the Bacteriophages

The bacterial killing activity was determined individually for each phage (ZCEC10, ZCEC11, and ZCEC12) at different MOIs (0.01, 0.1, 1, and 10) against *E. coli* O18, as previously described in [33], with slight modifications. Briefly, 20 μL aliquot of every single phage at 10^9^, 10^8^,10^7^, and 10^6^ PFU/mL was mixed separately with 180 μL of the bacterial host at a concentration of 10^7^ CFU/mL, giving MOIs of 10, 1, 0.1, and 0.01, respectively. Then, the mixtures were incubated at 37 °C, and the bacterial growth was determined by measuring OD_600_ using a microplate reader (FLUOstar Omega, BMG LABTECH, Ortenberg, Germany). Data were collected at 10 min intervals for 2.5 h using the MARS Data Analysis Software package (version 3.42). The bacterial culture without phage inoculation was used as a control. The experiments were performed in triplicate.

### 2.8. Examination of Phage Morphology by Transmission Electron Microscopy (TEM)

The size and morphology were determined using TEM available as a core facility at the Faculty of Science, Alexandria University (Alexandria, Egypt). The phage suspension was prepared in SM buffer with 10^10^ PFU/mL and placed in TEM (JEOL JEM-1400 Plus).

### 2.9. Phage DNA Sequencing and Bioinformatic Analysis

Phage DNA was extracted using the phenol/chloroform method as previously described [34]. The extracted DNA was diluted to 0.2 ng/µL and for whole-genome sequencing. Libraries were prepared using the Nextera XT Kit (Illumina, Cambridge, UK) following the manufacturer’s instructions. Final library insert sizes were determined using an Agilent Bioanalyzer 2000 and ranged between 200 and 1000 bp. Library concentrations were determined using the Qubit 3.0 and Qubit DNA High Sensitivity Kit. Libraries were finally pooled and sequenced on the illumina MiSeq platform and MiSeq Reagent Nano Kit V2 (2 X 150 bp). The sequenced reads were evaluated for accuracy using FASTQC [35]. Reads with low-quality were trimmed using PRINSEQ. Sequences were assembled de novo using SPAdes [36] with (K-mers: 21, 33, 55, 77, and 99). QUAST software was used to check the quality of the genome assembly [37]. The closely related phages were identified using BLASTn against the NCBI nucleotide database. Then, they were imported into MEGA-X [38] to draw a phylogenetic tree using the following default parameters: CLUSTAL-W aligner [39] and the maximum likelihood fit model. Open-reading frames were predicted using the ORF finder tool (https://www.ncbi.nlm.nih.gov/orffinder/; Access date: 27 July 2021) using methionine and alternative initiation codons as start codons. The putative coding sequences (CDSs) were predicted using BLASTp of the predicted ORFs against the NCBI non-redundant protein sequences (nr) database. To increase the confidence in the predicted ORFs and CDSs, they were compared to those predicted through PHASTER [40]. Genetic maps focusing on putative coding genes were generated using SNAPGene (GSL Biotech; available at https://www.snapgene.com/; Access date: 27 July 2021).

### 2.10. Accession Numbers

The complete annotated genome of phages ZCEC10, ZCEC11, and ZCEC12 has been deposited in the GenBank database under the accession numbers OK310512, OK310513, and OK310514, respectively.

#### Statistical Analysis

Data were collected and analyzed using GraphPad Prism version 9 software.

## 3. Results

### 3.1. Antimicrobial Susceptibility Testing

The susceptibility of the *E. coli* O18 strain to the tested antibiotics varied from sensitive, intermediate, and resistant, according to the diameter of the inhibition zone. The bacterial strain showed resistance to four antibiotics including ampicillin/sulbactam, ampicillin, gentamicin, and tetracycline. Moreover, it was intermediate resistant to two antibiotics: amoxicillin/clavulanic acid and amikacin (Table 1).

### 3.2. Isolation of Bacteriophages

Three phages identified as ZCEC10, ZCEC11, and ZCEC12 against *E. coli* O18 as a bacterial host were isolated from hospital sewage water samples in Egypt. Clear plaques appeared after 18 h incubation at 37 °C.

### 3.3. Host Range Determination

Five pathogenic *E. coli* reference strains (*E. coli* O157-H7, *E. coli* O2, *E. coli* O-127-H6, *E. coli* ATCC 8739, and *E. coli* O18 (indicator host)) showed susceptibility toward the individual phages, while other strains did not exhibit any clear zone as shown in Table 2.

### 3.4. Sensitivity of Virions to pH and Temperature

**Figure 1:**
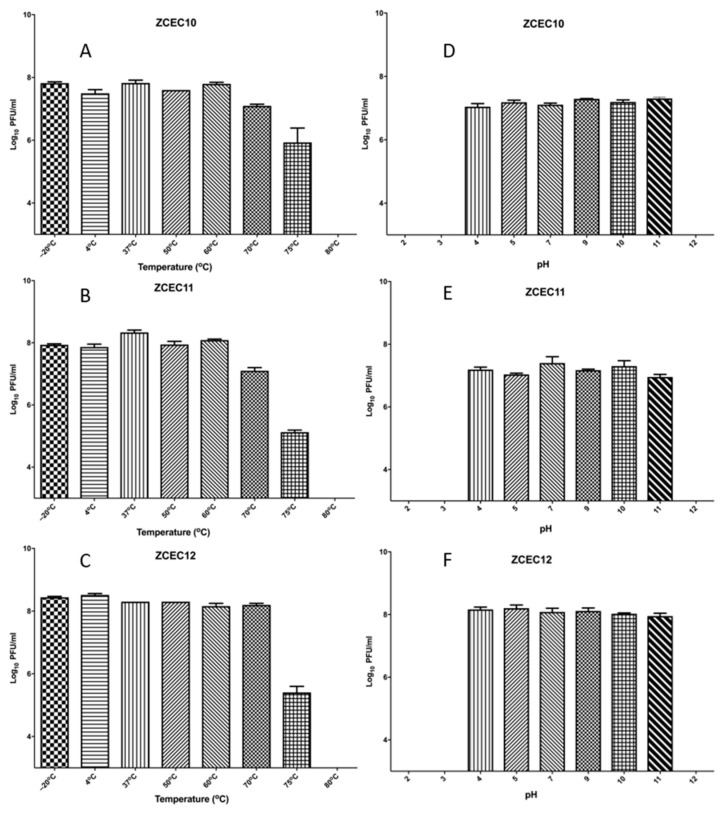
Stability of ZCEC10, ZCEC11, and ZCEC12 at different pH and temperatures. The figure represents the stability of phages at different temperatures: (**A**) ZCEC10, (**B**) ZCEC11, and (**C**) ZCEC12 and the stability of phages at different pH values: (**D**) ZCEC10, (**E**) ZCEC11, and (**F**) ZCEC12.

The titer of the three phages ZCEC10, ZCEC11, and ZCEC12 were stable at approximately 10^8^ PFU/mL for 60 min at temperatures of −20, 4, 37, 50, and 60 °C. However, when the phages were incubated at 70 °C, the titer was stable at 10^8^ PFU/mL for ZCEC12 and decreased to 10^7^ PFU/mL for both ZCEC10 and ZCEC11. At 75 °C incubation, the titer of the phages decreased rapidly to approximately 10^5^ PFU/mL. Upon incubation at 80 °C, all the titers drastically dropped below the detection limit. These results indicate that the phages under study could tolerate the standard environmental temperature. ZCEC12 remained viable at a pH range of 4.0–11.0 at approximately 10^8^ PFU/mL, while the titers of ZCEC10 and ZCEC11 decreased to 10^7^ PFU/mL. When incubated at pH 2, 3, and 12, the phages were utterly inactive. Thus, the optimum pH range for phages ZCEC10, ZCEC11, and ZCEC12 was found to be 4.0–11.0 (Figure 1).

### 3.5. Time Killing Curves

**Figure 2:**
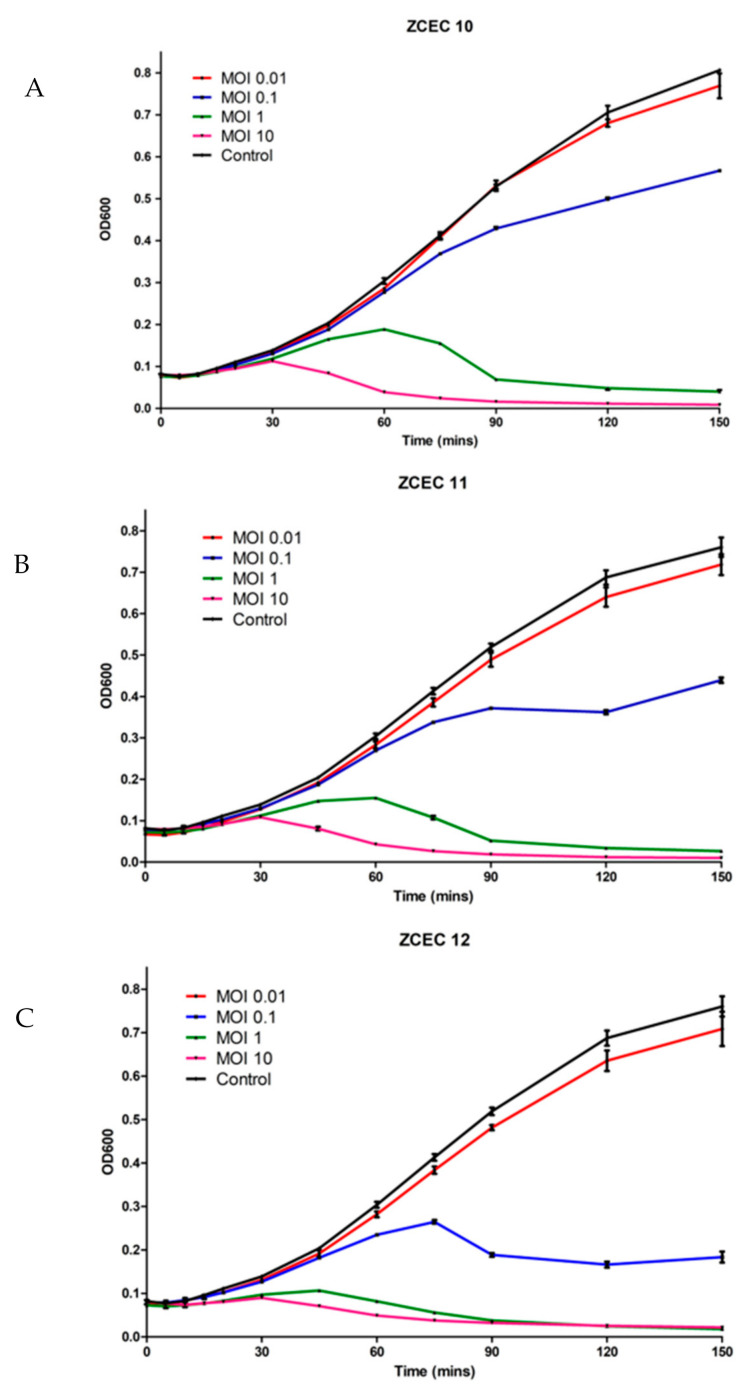
Time killing assay of phages against *E. coli* at 37 °C. These panels show the bacterial count of *E. coli* as the control and bacterial survival infected with three different phages: (**A**) ZCEC10; (**B**) ZCEC11; (**C**) ZCEC12 at different MOIs (0.01, 0.1, 1, and 10). Optical density at 600 nm was measured every 10 min up to 2.5 h. MOI: Multiplicity of infection, OD: Optical density.

*E. coli* O18 was cultured in TSB broth and infected with three distinct phages (ZCEC10, ZCEC11, and ZCEC12) using varied MOI values to test the phage’s killing activity on the host strain (0.01, 0.1, 1, and 10). After that, optical densities at OD600 were used to track bacterial development. In each case, phage infection resulted in bacterial growth inhibition, which became more pronounced as the MOI increased. On the *E. coli* O18 strain, the lysis kinetics of the three phages were measured. At all MOI values, the optical density of the culture declined around 90 min after infection. Due to the phage infection, the bacterial growth rate was lowered at a high phage titer (MOI of 10). Bacterial growth was better at a lower phage titer (MOI of 0.01) than infected cells at MOIs of 0.1, 1, and 10, but remained lower than the non-infected control (Figure 2). The non-infected culture represents the standard microbial growth of *E. coli* O18 over the incubation time.

### 3.6. Bioinformatics Analysis and Characterization of Bacteriophages Genome

**Figure 3:**
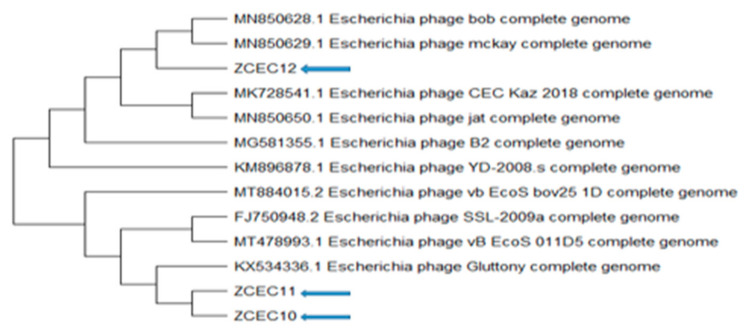
Phylogenetic relationships between the ZCEC10, ZCEC11, and ZCEC12 phages and BLASTN top-matched phages.

**Figure 4:**
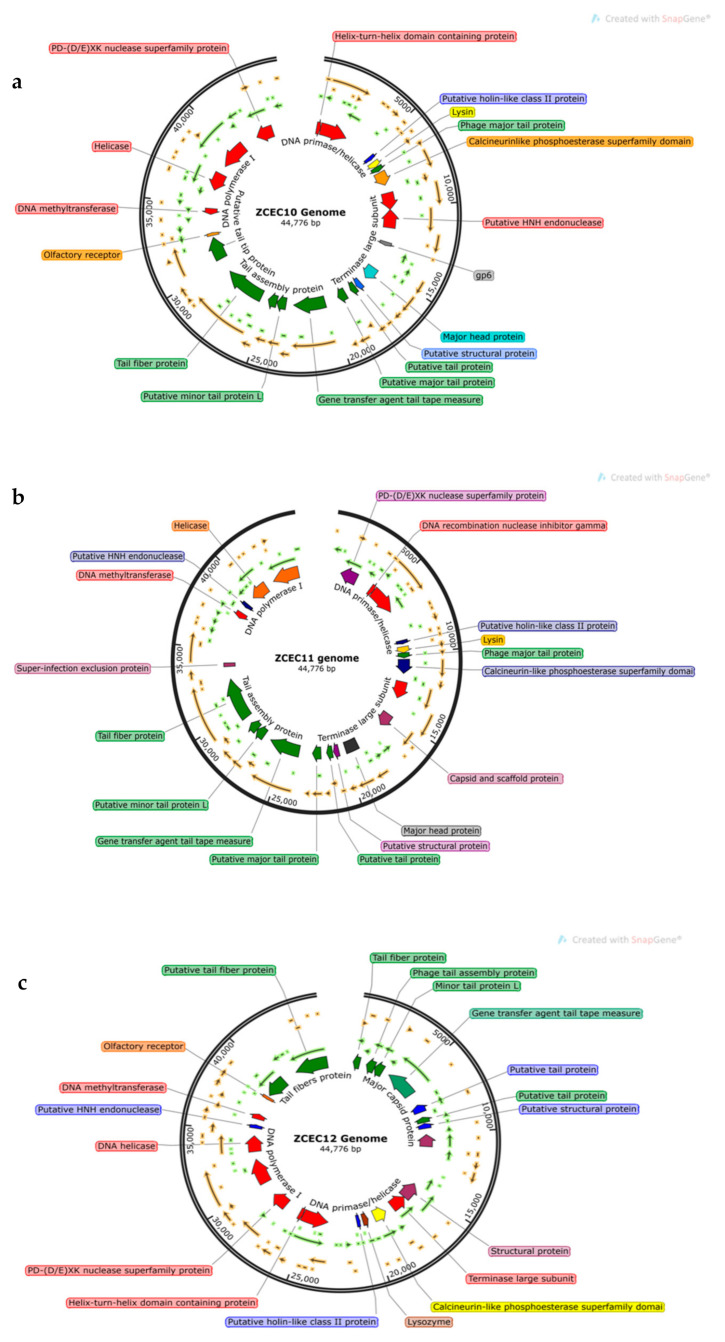
(**a**–**c**) Genetic maps of the three phages. Only functional genes are highlighted.

Through pulsed-field gel electrophoresis (PFGE), bacteriophages ZCEC10, ZCEC11, and ZCEC12 had a double-stranded DNA genome of around 45 kbp, which is equivalent to the values suggested by the International Committee on Taxonomy of Viruses (ICTV) for bacteriophages in the *Siphoviridae* family. The complete genomes of phage ZCEC10, ZCEC11, and ZCEC12 were sequenced and deposited in the NCBI GenBank database (GenBank Acc. No. OK310512, OK310513, and OK310514, respectively). Assembly quality for phages is summarized in Supplementary Table S1. BLASTn alignment and phylogenetic trees showed that the three phages were different from one another, as indicated in Figure 3. BLASTn analysis confirmed that the three phages were members of the *Siphoviridae* family, in the order Caudovirales. The annotated genes were manually curated and listed in Supplementary Tables S2–S4. The prediction of the open reading frames (ORFs) by applying the standard genetic code identified eighty-one putative protein-coding genes, among which thirty-six predicted proteins had assigned functions in ZCEC10. It had fifty-two ORFs on the leading strand and twenty-eight ORFs on the complementary strand. In addition, eighty-two putative protein-coding genes and thirty-four functional proteins were identified in ZCEC11, with fifty-four ORFs on the leading strand and twenty-eight ORFs on the complementary strand. ZCEC12 had eighty-one protein-coding genes and thirty-five functional proteins, with twenty-eight ORFs on the leading strand and fifty-four ORFs on the complementary strand. The functional genes of the three phages are presented in genetic maps (Figure 4). Functional proteins in the three phages had assigned functions such as cell lysis proteins, DNA replication/transcription/repair proteins, structural proteins, and DNA packaging proteins. None of the phages had any lysogenic genes (e.g., transposases, integrases, and prophages).

### 3.7. Bacteriophage Morphology

**Figure 5:**
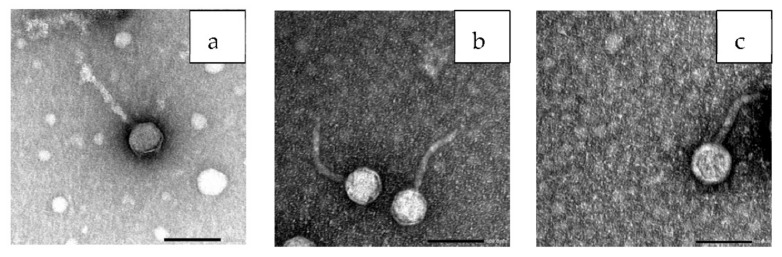
TEM images showing the morphology of the individual phages: (**a**) ZCEC10 phage, (**b**) ZCEC11 phage, and (**c**) ZCEC12 phage, scale bar represents 100 nm.

The electron micrograph revealed that the three phages under study had typical morphology of *Siphoviridae* family with an icosahedral head and long thin tail. Phage ZCEC10 had a head diameter with approximately 58 nm and the tail length of approximately 125 nm (Figure 5a), whereas phage ZCEC11 had a head diameter of approximately 65 nm and the tail length of approximately 122 nm (Figure 5b). The phage ZCEC12 head diameter was found to be approximately 70 nm with a long tail measuring approximately 130 nm in length (Figure 5c). Four phage particles were measured for ZCEC10 phage (Figure S1), and five phage particles were measured for both ZCEC11 and ZCEC12 phages (Figures S2 and S3).

## 4. Discussion

Due to excessive and imprudent use of antibiotics in human and veterinary medicine, the post antibiotic era is underway when all of our discovered antibiotics become outmaneuvered by multi-drug resistant bacteria (MDR) or superbugs [41]. Antimicrobial resistance (AMR), currently the second leading cause of deaths worldwide, killing 700.000 people a year, is expected to reach a mortality rate of 10 million deaths by the year 2050 and might even exceed cancer [41]. This necessitates conglomerate efforts toward the development of alternative therapies to antibiotics within a narrow time frame. Bacteriophage is the most promising alternative to antibiotics for treating infections due to ‘superbugs’ [42,43,44]. In this research study, three different bacteriophages were successfully isolated from hospital sewage samples against *E. coli* O18 as a bacterial host. The isolated phages were classified as members of the *Siphoviridae* family with an icosahedral head and long thin tail. In a study conducted by Nishikawa et al., T4 and T6 lytic phages had a limited hosting range, but phage KEP10 showed lytic activity against a wide host range [45]. In our study, phages ZCEC10, ZCEC11, and ZCEC12 showed lytic activity against the highly pathogenic bacterial strains including *E. coli* O157-H7, *E. coli* O2, *E. coli* O-127-H6, *E. coli* ATCC 8739, and *E. coli* O18. The stability of the phages at a broader pH range and temperature is critical for preservation and in clinical use as phage therapy [46]. Several studies have reported that bacteriophages may differ in both their thermal and pH stability [47]. In the current study, phages ZCEC10, ZCEC11, and ZCEC12 maintained a high viability in the pH range of 4.0–11.0 under physiological conditions, which is in agreement with previous studies [29,48,49]. However, in another study, the titer of VB_EcoS-Golestan phage was active and stable only at pH values of 7.0 and 8.0 [50]. Interestingly, the ZCEC10, ZCEC11, and ZCEC12 phages also showed excellent thermal stability from −20 °C to 70 °C, were slightly active at 75 °C, and inactive at 80 °C. These results showed that all phages were thermally stable since the phage particles were observed after incubation [51]. Previous studies revealed that an increase in temperature decreases the titer of phages [52]. However, numerous phages that have been studied thus far can survive high temperatures [53]. When phages are used to challenge bacterial cells, the multiplicity of infection is an important parameter to consider. In this study, when bacteria were infected with phage at lower MOIs (0.01 and 0.1), the bacterial density (OD600) rapidly increased, whereas when bacteria were inactivated to bacteria cells earlier than at higher MOIs, the bacterial density (OD600) rapidly decreased (1.0 and 10). Furthermore, a comparison of the killing curves of the three isolated phages against the host at the tested MOIs showed that optimal MOI was 10 because of the reduction in the viable bacterial count. The results showed that the efficacy of phages to control the bacterial host *E. coli* O18 is concentration dependent. This observation was identified in previous studies when both phages vB_KpnS_Kp13 and ZCKP8 were subjected to *K. pneumoniae* in a concentration dependent manner in LB and TSB broth over hours [29,54]. Moreover, whole-genome sequence analysis and functional annotation showed that phages ZCEC10, ZCEC11, and ZCEC12 did not encode any genes related to virulence and antibiotic resistance genes, supporting that the isolated phages might be promising therapeutic agents to control *E. coli* O18 infections in poultry.

## Figures and Tables

**Table 1:** Phenotypic sensitivity of *E. coli* O18 strain to ten different antibiotics.

Class	Antibiotic Name	Abbreviation	Diameter (mm)	Susceptibility
Aminoglycoside	Gentamicin	CN 10	7	Resistant
Beta-lactam antibiotics (monobactam)	Aztreonam	ATM 30	24	Sensitive
Penicillin-like antibiotics	Amoxicillin/Clavulanic acid	AMC 30	16	Intermediate
Beta-lactam antibiotics (First generation)	Ampicillin	AMP 10	11	Resistant
Aminoglycoside	Amikacin	AK 30	16	Intermediate
Cephalosporin (second generation)	Cefoxitin	FOX 30	18	Sensitive
Pencillins/inhibitor combination	Ampicillin/sulbactam	SAM 20	0	Resistant
Cephalosporin (third generation)	Cefotaxime	CTX 30	28	Sensitive
Tetracycline	Tetracycline	TE 30	0	Resistant
Cephalosporin (third generation)	Ceftriaxone	CRO 30	25	Sensitive

**Table 2:** Shows the bacterial strain *E. coli* O18 and their susceptibility to phages.

Bacterial Strain	ZCEC10	ZCEC11	ZCEC12
*E. coli* O157-H7	+	+	+
*E. coli* O2	+	+	+
*E. coli* O-127-H6	+	+	+
*E. coli*- ATCC 8739	+	+	+
*Salmonella enterica* Typhimurium- ATCC 14028	−	−	−
*Salmonella* Kent	−	−	−
*E. coli* O25	−	−	−
*E. coli* O88	−	−	−
*E. coli* O78	−	−	−
*E. coli* O18	+	+	+
*E. coli* O1	−	−	−
*E. coli* O12	−	−	−
*E. coli* O6	−	−	−
*E. coli* O186	−	−	−
*E. coli* O-hemolytic	−	−	−
*E. coli* O125	−	−	−
*E. coli* O55	−	−	−
*E. coli* O125	−	−	−
*E. coli* O115	−	−	−
*E. coli* O27	−	−	−
*E. coli* O168	−	−	−
*E. coli* O164	−	−	−
*E. coli* O114	−	−	−
*E. coli* O151	−	−	−
*E. coli* O169	−	−	−

(+) indicates clear lysis, and (−) indicates no lysis.

## Data Availability

The dataset presented in this study can be found in online repositories. The genome sequences were deposited in GenBank under the accession numbers OK310512, OK310513, and OK310514.

## Supplementary Materials

The following supporting information can be downloaded at: https://www.mdpi.com/article/10.3390/microorganisms10030589/s1. Figure S1: TEM images showing the morphology of the ZCEC10 phage particles, Figure S2: TEM images showing the morphology of the ZCEC11 phage particles, Figure S3: TEM images showing the morphology of the ZCEC12 phage particles. Table S1: Assembly quality report for the three phages, Table S2: Genome annotation of the ZCEC10 genome, Table S3: Genome annotation of the ZCEC11 genome, Table S4: Genome annotation of the ZCEC12 genome.
