# Supplementary material for: Isolation, Characterization, and Genomic Analysis of Three Novel E. coli Bacteriophages That Effectively Infect E. coli O18
Source: Microorganisms. 2022 Mar 9;10(3):589. doi: 10.3390/microorganisms10030589 (PMC8954371; doi:10.3390/microorganisms10030589)
Supplement: Supplementary file 1 [file microorganisms-10-00589-s001.zip › microorganisms-1558432-supplementary.pdf]

Figure S1. TEM images showing the morphology of the ZCEC10 phage particles.

ZCEC10 Phage

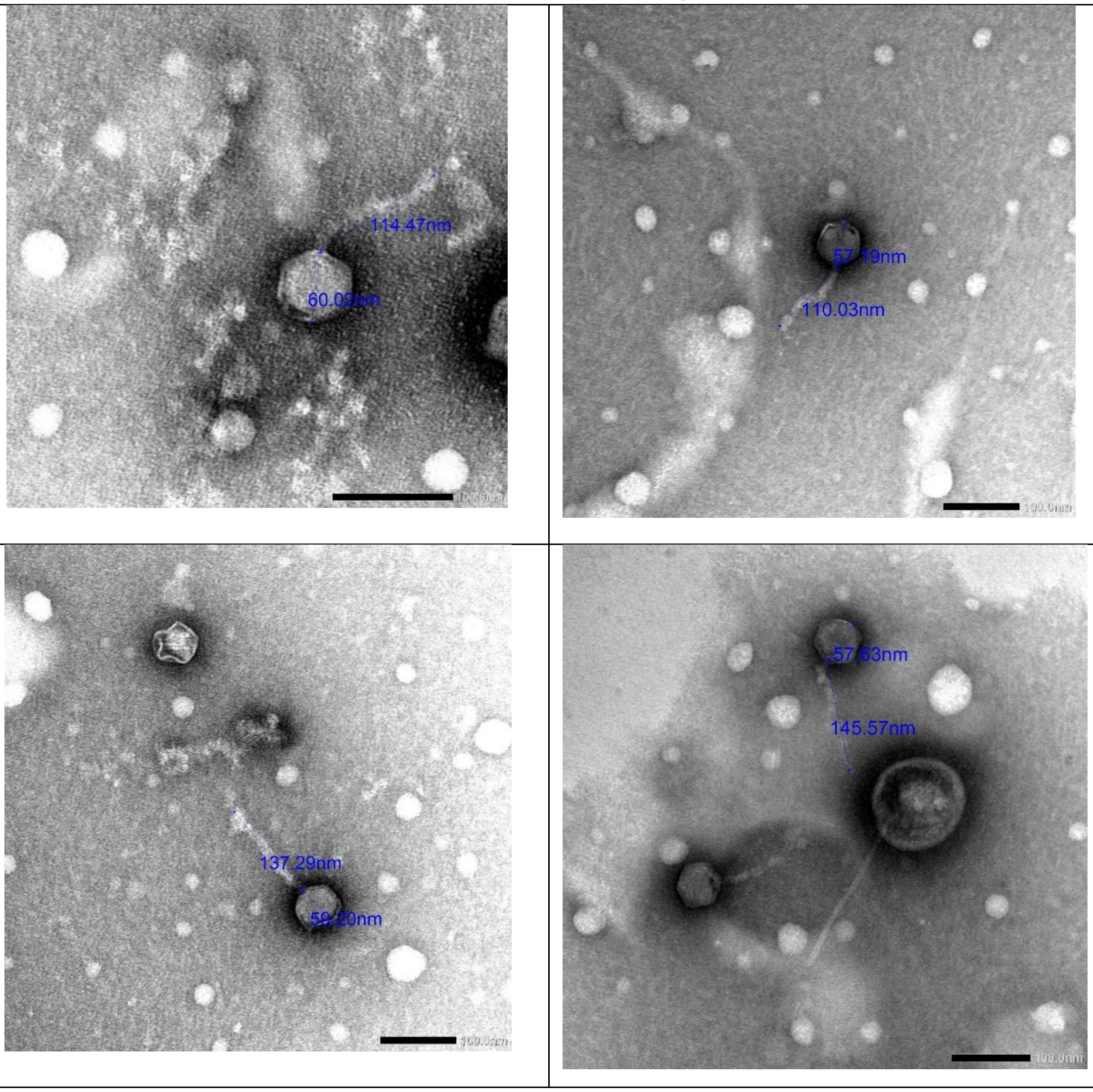

Average head size=(60.08+57.19+57.63+59.2)/4= **58.5**
Average tail size=(114.7+110+137.29+145.5)/4= **126.87**

Figure S2: TEM images showing the morphology of the ZCEC11phage particles.

<table>
<tr><td colspan="2">ZCEC11 Phage</td></tr>
<tr><td>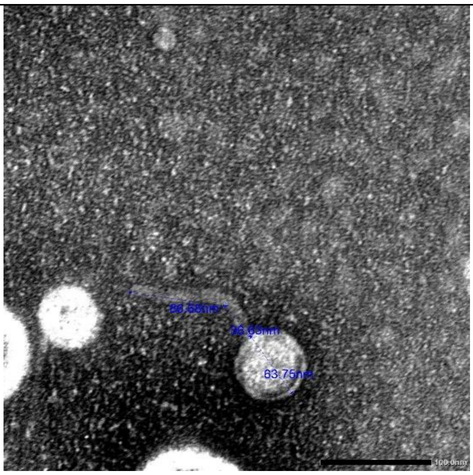</td><td>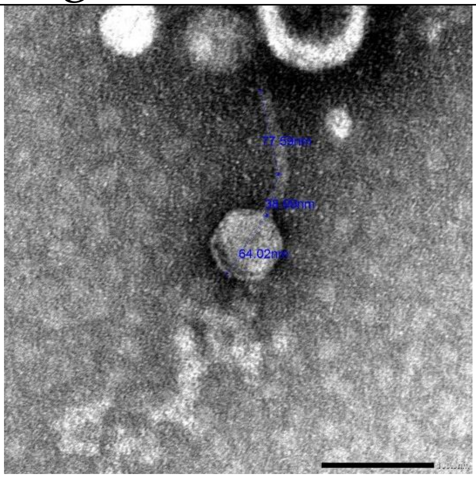</td></tr>
<tr><td>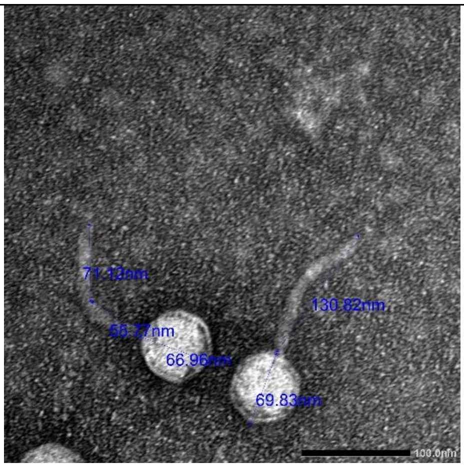</td><td>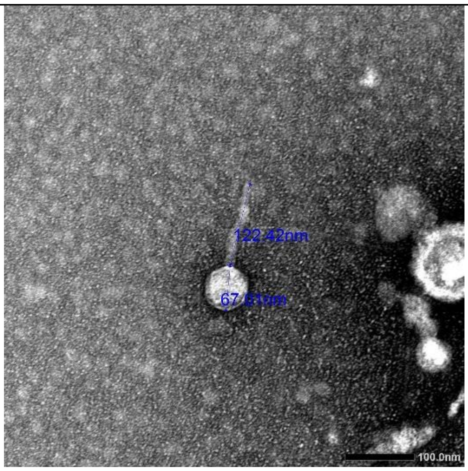</td></tr>
<tr><td colspan="2">Average head size=(63.7+64.02+69.8+66.9+67)/5=66.28
Average tail size=(123.28+116.58+130.8+126.87+122.4)/5=123.98</td></tr>
</table>

Figure S3: TEM images showing the morphology of the ZCEC12 phage particles.

ZCEC12 Phage

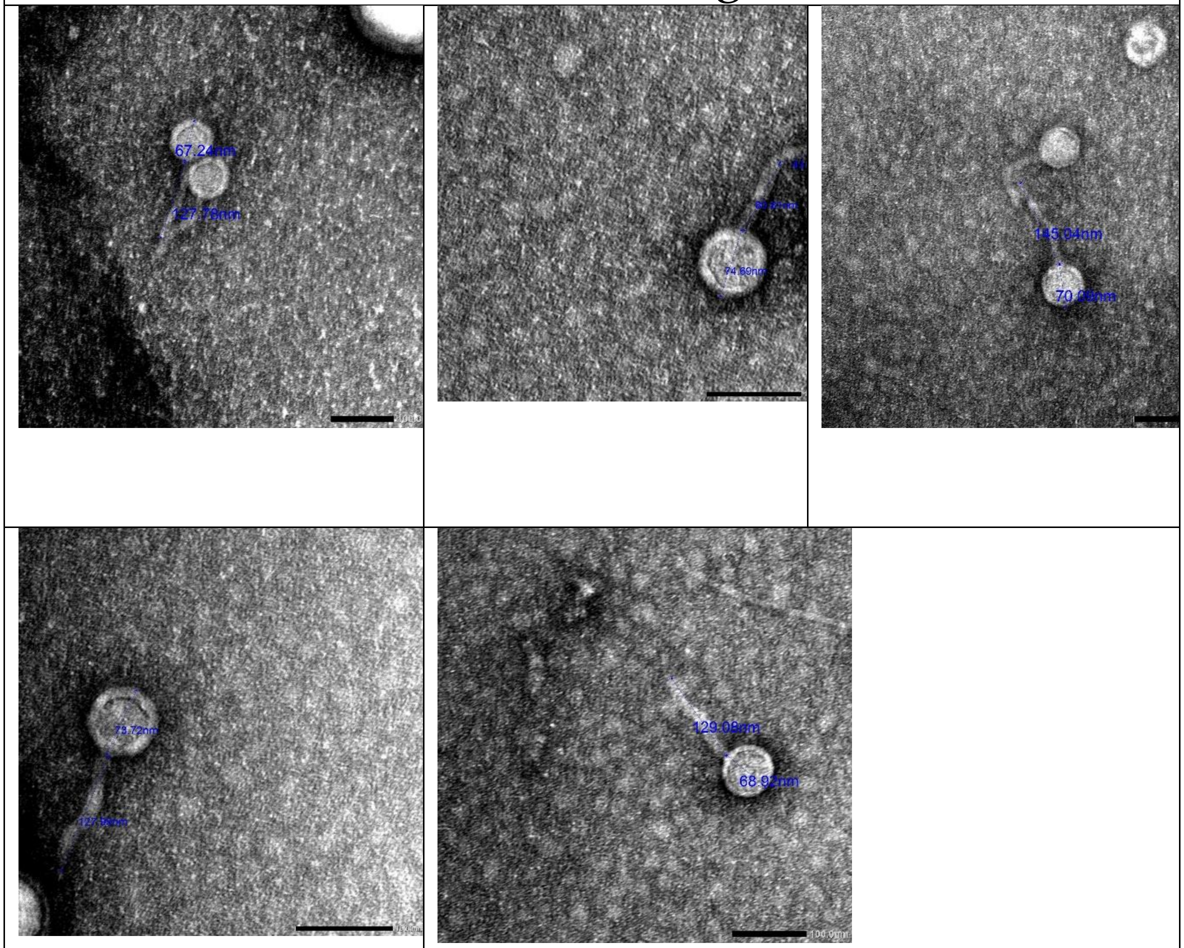

Average head size=(67.24+70.09+74.7+73.7+68.9)/5=**70.92**
Average tail size=(127.78+145.04+124.4+127.99+129)/5=**130.84**

Table S1. Assembly quality report for the three phages

Assembly parameters	ZCEC10	ZCEC11	ZCEC12
# contigs (>= 0 bp	203	130	82
# contigs (>= 1000 bp	12	2	1
# contigs (>= 5000 bp	1	1	1
# contigs (>= 10000 bp	1	1	1
# contigs (>= 25000 bp	1	1	1
# contigs (>= 50000 bp	0	0	0
Total length (>= 0 bp	118573	87109	69224
Total length (>= 1000 bp	60644	46047	44776
Total length (>= 5000 bp	44776	44776	44776
Total length (>= 10000 bp	44776	44776	44776
Total length (>= 25000 bp	44776	44776	44776
Total length (>= 50000 bp	0	0	0
# contigs	23	15	3
Largest contig	44776	44776	44776
Total length	68068	53494	46183
GC (%	52.38	53.3	54.57
N50	44776	44776	44776
N75	1459	44776	44776
L50	1	1	1
L75	5	1	1
# N's per 100 kbp	0	0	0

Table S2. Genome annotation of the ZCEC10 genome

ORF number	Strand	Frame	CDS position	Translation	Best annotated protein
ORF10	+	1	5569 .. 5880	312 | 103	Putative holin-like class I protein
ORF100	+	2	4238 .. 4780	543 | 180	Hypothetical Protein
ORF102	+	2	5348 .. 5638	291 | 96	Putative holin-like class II protein
ORF103	+	2	5867 .. 6358	492 | 163	Lysin
ORF111	+	2	8075 .. 8725	651 | 216	Putative terminase small subunit
ORF120	+	2	12692 .. 13051	360 | 119	Hypothetical Protein
ORF124	+	2	14225 .. 15154	930 | 309	Hypothetical Protein
ORF137	+	2	20162 .. 20248	87 | 28	Hypothetical Protein
ORF138	+	2	20321 .. 22963	2643 | 880	Gene transfer agent tail tape measure
ORF14	+	1	6901 .. 8043	1143 | 380	Calcineurin-like phosphoesterase superfamily domain protein
ORF141	+	2	24386 .. 25126	741 | 246	Tail assembly protein
ORF16	+	1	8725 .. 10110	1386 | 461	Terminase large subunit
ORF187	+	3	4863 .. 4973	111 | 36	Hypothetical Protein
ORF190	+	3	5559 .. 5687	129 | 42	Hypothetical Protein
ORF194	+	3	6375 .. 6782	408 | 135	Phage major tail protein
ORF198	+	3	10122 .. 11642	1521 | 506	Structural protein
ORF2	+	1	235 .. 564	330 | 109	Helix-turn-helix domain containing protein
ORF20	+	1	11626 .. 12729	1104 | 367	Capsid and scaffold protein
ORF200	+	3	12858 .. 13163	306 | 101	gp6
ORF205	+	3	15252 .. 16352	1101 | 366	Major head protein
ORF206	+	3	16920 .. 17384	465 | 154	Putative structural protein
ORF208	+	3	17979 .. 18401	423 | 140	Putative tail completion protein
ORF209	+	3	18462 .. 19187	726 | 241	Putative major tail protein
ORF21	+	1	13264 .. 13395	132 | 43	Hypothetical Protein
ORF210	+	3	19824 .. 19937	114 | 37	Hypothetical Protein
ORF215	+	3	23595 .. 24383	789 | 262	Putative minor tail protein L
ORF219	+	3	25731 .. 29150	3420 | 1139	Tail fiber protein

ORF222	+	3	29493 .. 30164	672 | 223	Hypothetical Protein
ORF224	+	3	30423 .. 32315	1893 | 630	Putative tail tip protein
ORF229	+	3	34110 .. 34262	153 | 50	Hypothetical Protein
ORF240	+	3	38946 .. 39386	441 | 146	Hypothetical Protein
ORF241	+	3	39975 .. 40196	222 | 73	Putative DNA polymerase 1
ORF241	+	3	39975 .. 40196	222 | 73	Putative DNA polymerase 1
ORF242	+	3	40404 .. 40985	582 | 193	Putative DNA polymerase 1
ORF250	-	1	44710 .. 44018	693 | 230	Hypothetical Protein
ORF251	-	1	43969 .. 43718	252 | 83	Hypothetical Protein
ORF256	-	1	41392 .. 40769	624 | 207	Hypothetical Protein
ORF268	-	1	37960 .. 37730	231 | 76	Hypothetical Protein
ORF27	+	1	16369 .. 16911	543 | 180	Hypothetical Protein
ORF270	-	1	36301 .. 35591	711 | 236	Putative DNA cytosine methyltransferase C5
ORF272	-	1	35299 .. 34724	576 | 191	Hypothetical Protein
ORF29	+	1	17476 .. 17979	504 | 167	Putative tail protein
ORF317	-	1	19810 .. 19643	168 | 55	Hypothetical Protein
ORF335	-	1	14440 .. 14288	153 | 50	Hypothetical Protein
ORF34	+	1	19216 .. 19623	408 | 135	Hypothetical Protein
ORF369	-	2	43539 .. 42109	1431 | 476	PD-(D/E)XK nuclease superfamily protein
ORF37	+	1	20245 .. 20358	114 | 37	Hypothetical Protein
ORF370	-	2	38568 .. 38203	366 | 121	Putative olliday junction resolvase
ORF371	-	2	37752 .. 36298	1455 | 484	Helicase
ORF377	-	2	34737 .. 34252	486 | 161	DNA methyltransferase
ORF379	-	2	34050 .. 33580	471 | 156	Hypothetical Protein
ORF38	+	1	22960 .. 23598	639 | 212	Phage minor tail protein
ORF380	-	2	33516 .. 32962	555 | 184	Hypothetical Protein
ORF381	-	2	32919 .. 32344	576 | 191	Hypothetical Protein
ORF418	-	2	14709 .. 13981	729 | 242	Hypothetical Protein
ORF419	-	2	13941 .. 13177	765 | 254	Hypothetical Protein
ORF43	+	1	25117 .. 25734	618 | 205	Putative tail assembly protein
ORF435	-	2	7905 .. 7744	162 | 53	Hypothetical Protein
ORF437	-	2	7581 .. 7297	285 | 94	Hypothetical Protein
ORF461	-	3	43799 .. 43539	261 | 86	Hypothetical Protein
ORF467	-	3	42116 .. 41874	243 | 80	Hypothetical Protein
ORF468	-	3	41798 .. 40998	801 | 266	Hypothetical Protein
ORF470	-	3	40772 .. 38481	2292 | 763	DNA polymerase I
ORF471	-	3	38222 .. 37989	234 | 77	Hypothetical Protein
ORF477	-	3	35594 .. 35271	324 | 107	Putative HNH endonuclease
ORF479	-	3	34271 .. 34047	225 | 74	Hypothetical Protein
ORF490	-	3	30464 .. 30183	282 | 93	Super-infection exclusion protein
ORF5	+	1	3190 .. 3273	84 | 27	Hypothetical Protein
ORF54	+	1	29098 .. 29490	393 | 130	Hypothetical Protein
ORF568	-	3	3308 .. 3165	144 | 47	Hypothetical Protein
ORF6	+	1	3496 .. 3801	306 | 101	Hypothetical Protein
ORF63	+	1	32482 .. 32724	243 | 80	Olfactory receptor
ORF65	+	1	32845 .. 33351	507 | 168	Hypothetical Protein
ORF66	+	1	33367 .. 33564	198 | 65	Hypothetical Protein
ORF67	+	1	33583 .. 34008	426 | 141	Hypothetical Protein
ORF7	+	1	3811 .. 4080	270 | 89	Hypothetical Protein
ORF8	+	1	4777 .. 4917	141 | 46	Hypothetical Protein
ORF9	+	1	4996 .. 5295	300 | 99	Hypothetical Protein
ORF96	+	2	575 .. 2839	2265 | 754	DNA primase/helicase
ORF97	+	2	2843 .. 3229	387 | 128	Hypothetical Protein

ORF98	+	2	3260 .. 3499	240 | 79	Hypothetical Protein

Table S3. Genome annotation of the ZCEC11 genome

ORF number	Strand	Frame	CDS position	Translation	Best annotated protein
ORF101	+	2	8423 .. 8533	111 | 36	Hypothetical protein
ORF104	+	2	9119 .. 9247	129 | 42	Hypothetical protein
ORF108	+	2	9935 .. 10342	408 | 135	Phage major tail protein
ORF11	+	1	4135 .. 6399	2265 | 754	DNA primase/helicase
ORF112	+	2	13682 .. 15202	1521 | 506	Structural protein
ORF114	+	2	16418 .. 16723	306 | 101	Hypothetical protein
ORF119	+	2	18812 .. 19912	1101 | 366	Major head protein
ORF12	+	1	6403 .. 6789	387 | 128	Hypothetical protein
ORF120	+	2	20480 .. 20944	465 | 154	Putative structural protein
ORF122	+	2	21539 .. 21961	423 | 140	Tc1 tail completion protein
ORF123	+	2	22022 .. 22747	726 | 241	Putative major tail protein
ORF124	+	2	23384 .. 23497	114 | 37	Hypothetical protein
ORF129	+	2	27155 .. 27943	789 | 262	Putative minor tail protein L
ORF13	+	1	6820 .. 7059	240 | 79	Hypothetical protein
ORF133	+	2	29291 .. 32710	3420 | 1139	Tail fiber protein
ORF136	+	2	33053 .. 33724	672 | 223	Hypothetical protein
ORF138	+	2	33983 .. 35875	1893 | 630	Tail fibers protein
ORF138	+	2	33983 .. 35875	1893 | 630	Tail fibers protein
ORF143	+	2	37670 .. 37822	153 | 50	Hypothetical protein
ORF15	+	1	7798 .. 8340	543 | 180	Hypothetical protein
ORF154	+	2	42506 .. 42946	441 | 146	Hypothetical protein
ORF155	+	2	43535 .. 43756	222 | 73	Putative DNA polymerase 1
ORF155	+	2	43535 .. 43756	222 | 73	Putative DNA polymerase 1
ORF156	+	2	43964 .. 44545	582 | 193	Hypothetical protein
ORF164	+	3	3795 .. 4124	330 | 109	DNA recombination nuclease inhibitor gamma
ORF167	+	3	6750 .. 6833	84 | 27	Hypothetical protein
ORF168	+	3	7056 .. 7361	306 | 101	Hypothetical protein
ORF169	+	3	7371 .. 7640	270 | 89	Hypothetical protein
ORF17	+	1	8908 .. 9198	291 | 96	Putative holin-like class II protein
ORF170	+	3	8337 .. 8477	141 | 46	Hypothetical protein
ORF171	+	3	8556 .. 8855	300 | 99	Hypothetical protein
ORF172	+	3	9129 .. 9440	312 | 103	Putative holin-like class I protein
ORF176	+	3	10461 .. 11603	1143 | 380	Calcineurin-like phosphoesterase superfamily domain protein
ORF178	+	3	12285 .. 13670	1386 | 461	Terminase large subunit
ORF18	+	1	9427 .. 9918	492 | 163	Lysin
ORF182	+	3	15186 .. 16289	1104 | 367	Capsid and scaffold protein
ORF183	+	3	16824 .. 16955	132 | 43	Hypothetical protein
ORF189	+	3	19929 .. 20471	543 | 180	Hypothetical protein
ORF191	+	3	21036 .. 21539	504 | 167	Putative tail protein
ORF196	+	3	22776 .. 23183	408 | 135	Hypothetical protein
ORF199	+	3	23805 .. 23918	114 | 37	Hypothetical protein
ORF200	+	3	26520 .. 27158	639 | 212	Phage minor tail protein
ORF205	+	3	28677 .. 29294	618 | 205	Putative tail assembly protein
ORF216	+	3	32658 .. 33050	393 | 130	Hypothetical protein
ORF225	+	3	36042 .. 36284	243 | 80	Hypothetical protein
ORF227	+	3	36405 .. 36911	507 | 168	Hypothetical protein
ORF228	+	3	36927 .. 37124	198 | 65	Hypothetical protein
ORF229	+	3	37143 .. 37568	426 | 141	Hypothetical protein

ORF250	-	1	44332 .. 42041	2292 | 763	DNA polymerase I
ORF251	-	1	41782 .. 41549	234 | 77	Hypothetical protein
ORF257	-	1	39154 .. 38831	324 | 107	Putative HNH endonuclease
ORF259	-	1	37831 .. 37607	225 | 74	Hypothetical protein
ORF26	+	1	11635 .. 12285	651 | 216	Terminase large subunit
ORF270	-	1	34024 .. 33743	282 | 93	Super-infection exclusion protein
ORF348	-	1	6868 .. 6725	144 | 47	Hypothetical protein
ORF35	+	1	16252 .. 16611	360 | 119	Hypothetical protein
ORF357	-	1	3571 .. 2879	693 | 230	Hypothetical protein
ORF358	-	1	2830 .. 2579	252 | 83	Hypothetical protein
ORF364	-	2	44727 .. 44329	399 | 132	Hypothetical protein
ORF376	-	2	41520 .. 41290	231 | 76	Hypothetical protein
ORF378	-	2	39861 .. 39151	711 | 236	C-5 cytosine-specific DNA methylase
ORF380	-	2	38859 .. 38284	576 | 191	Hypothetical protein
ORF39	+	1	17785 .. 18714	930 | 309	Hypothetical protein
ORF425	-	2	23370 .. 23203	168 | 55	Hypothetical protein
ORF443	-	2	18000 .. 17848	153 | 50	Hypothetical protein
ORF476	-	2	2400 .. 970	1431 | 476	PD-(D/E)XK nuclease superfamily protein
ORF477	-	3	42128 .. 41763	366 | 121	Putative restriction-endonuclease
ORF478	-	3	41312 .. 39858	1455 | 484	Helicase
ORF484	-	3	38297 .. 37812	486 | 161	DNA methyltransferase
ORF486	-	3	37610 .. 37140	471 | 156	Hypothetical protein
ORF487	-	3	37076 .. 36522	555 | 184	Hypothetical protein
ORF488	-	3	36479 .. 35904	576 | 191	Hypothetical protein
ORF52	+	1	23722 .. 23808	87 | 28	Hypothetical protein
ORF525	-	3	18269 .. 17541	729 | 242	Hypothetical protein
ORF526	-	3	17501 .. 16737	765 | 254	Hypothetical protein
ORF53	+	1	23881 .. 26523	2643 | 880	Gene transfer agent tail tape measure
ORF542	-	3	11465 .. 11304	162 | 53	Hypothetical protein
ORF544	-	3	11141 .. 10857	285 | 94	Hypothetical protein
ORF56	+	1	27946 .. 28686	741 | 246	Tail assembly protein
ORF568	-	3	2660 .. 2400	261 | 86	Hypothetical protein
ORF574	-	3	977 .. 735	243 | 80	Hypothetical protein
ORF575	-	3	659 .. >3	657 | 218	Hypothetical protein

Table S4. Genome annotation of the ZCEC12 genome

ORF number	Strand	Frame	CDS position	Translation	Best annotated protein
ORF175	+	2	23000 .. 23143	144 | 47	Hypothetical protein
ORF184	+	2	26297 .. 26989	693 | 230	Hypothetical protein
ORF185	+	2	27038 .. 27289	252 | 83	Hypothetical protein
ORF190	+	2	29615 .. 30238	624 | 207	Hypothetical protein
ORF202	+	2	33047 .. 33277	231 | 76	Hypothetical protein
ORF204	+	2	34706 .. 35416	711 | 236	Putative DNA cytosine methyltransferase C5
ORF206	+	2	35708 .. 36283	576 | 191	Hypothetical protein
ORF25	+	1	11599 .. 12327	729 | 242	Hypothetical protein
ORF252	+	3	6498 .. 6665	168 | 55	Hypothetical protein
ORF26	+	1	12367 .. 13131	765 | 254	Hypothetical protein
ORF270	+	3	11868 .. 12020	153 | 50	Hypothetical protein
ORF303	+	3	27468 .. 28898	1431 | 476	PD-(D/E)XK nuclease superfamily protein
ORF304	+	3	32439 .. 32804	366 | 121	Putative restriction-endonuclease
ORF305	+	3	33255 .. 34709	1455 | 484	DNA helicase
ORF311	+	3	36270 .. 36755	486 | 161	DNA methyltransferase
ORF313	+	3	36957 .. 37427	471 | 156	Hypothetical protein

ORF314	+	3	37491 .. 38045	555 | 184	Hypothetical protein
ORF315	+	3	38088 .. 38663	576 | 191	Hypothetical protein
ORF371	-	1	21445 .. 21335	111 | 36	Hypothetical protein
ORF374	-	1	20749 .. 20621	129 | 42	Hypothetical protein
ORF378	-	1	19933 .. 19526	408 | 135	Hypothetical protein
ORF382	-	1	16186 .. 14666	1521 | 506	Structural protein
ORF384	-	1	13450 .. 13145	306 | 101	Hypothetical protein
ORF389	-	1	11056 .. 9956	1101 | 366	Major capsid protein
ORF390	-	1	9388 .. 8924	465 | 154	Putative structural protein
ORF392	-	1	8329 .. 7907	423 | 140	Putative tail completion protein
ORF393	-	1	7846 .. 7121	726 | 241	Putative tail protein
ORF394	-	1	6484 .. 6371	114 | 37	Hypothetical protein
ORF399	-	1	2713 .. 1925	789 | 262	Minor tail protein L
ORF403	-	1	577 .. >2	576 | 191	Tail fiber protein
ORF403	-	1	577 .. >2	576 | 191	Tail fiber protein
ORF404	-	2	44763 .. 41857	2907 | 968	Putative tail fiber protein
ORF404	-	2	44763 .. 41857	2907 | 968	Tail fiber protein
ORF407	-	2	41514 .. 40843	672 | 223	Hypothetical protein
ORF409	-	2	40584 .. 38692	1893 | 630	Tail fibers protein
ORF414	-	2	36897 .. 36745	153 | 50	Hypothetical protein
ORF42	+	1	18403 .. 18564	162 | 53	Hypothetical protein
ORF425	-	2	32061 .. 31621	441 | 146	Hypothetical protein
ORF426	-	2	31032 .. 30811	222 | 73	Putative DNA polymerase 1
ORF435	-	2	26073 .. 25744	330 | 109	Helix-turn-helix domain containing protein
ORF438	-	2	23118 .. 23035	84 | 27	Hypothetical protein
ORF439	-	2	22812 .. 22507	306 | 101	Hypothetical protein
ORF44	+	1	18727 .. 19011	285 | 94	Hypothetical protein
ORF440	-	2	22497 .. 22228	270 | 89	Hypothetical protein
ORF441	-	2	21531 .. 21391	141 | 46	Hypothetical protein
ORF442	-	2	21312 .. 21013	300 | 99	Hypothetical protein
ORF443	-	2	20739 .. 20428	312 | 103	Putative holin-like class I protein
ORF447	-	2	19407 .. 18265	1143 | 380	Calcineurin-like phosphoesterase superfamily domain protein
ORF449	-	2	17583 .. 16198	1386 | 461	Terminase large subunit
ORF453	-	2	14682 .. 13579	1104 | 367	Capsid and scaffold protein
ORF454	-	2	13044 .. 12913	132 | 43	Hypothetical protein
ORF460	-	2	9939 .. 9397	543 | 180	Hypothetical protein
ORF462	-	2	8832 .. 8329	504 | 167	Putative tail protein
ORF467	-	2	7092 .. 6685	408 | 135	Hypothetical protein
ORF470	-	2	6063 .. 5950	114 | 37	Hypothetical protein
ORF471	-	2	3348 .. 2710	639 | 212	Phage minor tail protein
ORF476	-	2	1191 .. 574	618 | 205	Putative tail assembly protein
ORF487	-	3	41909 .. 41517	393 | 130	Hypothetical protein
ORF496	-	3	38525 .. 38283	243 | 80	Olfactory receptor
ORF498	-	3	38162 .. 37656	507 | 168	Hypothetical protein
ORF499	-	3	37640 .. 37443	198 | 65	Hypothetical protein
ORF500	-	3	37424 .. 36999	426 | 141	Hypothetical protein
ORF529	-	3	25733 .. 23469	2265 | 754	DNA primase/helicase
ORF530	-	3	23465 .. 23079	387 | 128	Hypothetical protein
ORF531	-	3	23048 .. 22809	240 | 79	Hypothetical protein
ORF533	-	3	22070 .. 21528	543 | 180	Hypothetical protein
ORF535	-	3	20960 .. 20670	291 | 96	Putative holin-like class II protein
ORF536	-	3	20441 .. 19950	492 | 163	Lysozyme

ORF544	-	3	18233 .. 17583	651 | 216	Terminase large subunit
ORF553	-	3	13616 .. 13257	360 | 119	Hypothetical protein
ORF557	-	3	12083 .. 11154	930 | 309	Hypothetical protein
ORF570	-	3	6146 .. 6060	87 | 28	Hypothetical protein
ORF571	-	3	5987 .. 3345	2643 | 880	Gene transfer agent tail tape measure
ORF574	-	3	1922 .. 1182	741 | 246	Phage tail assembly protein
ORF68	+	1	27208 .. 27468	261 | 86	Hypothetical protein
ORF74	+	1	28891 .. 29133	243 | 80	Hypothetical protein
ORF75	+	1	29209 .. 30009	801 | 266	Hypothetical protein
ORF77	+	1	30235 .. 32526	2292 | 763	DNA polymerase I
ORF78	+	1	32785 .. 33018	234 | 77	Hypothetical protein
ORF84	+	1	35413 .. 35736	324 | 107	Putative HNH endonuclease
ORF86	+	1	36736 .. 36960	225 | 74	Hypothetical protein
ORF97	+	1	40543 .. 40824	282 | 93	Super-infection exclusion protein
